# Dynamical Systems Analysis of Mitochondrial BAK Activation Kinetics Predicts Resistance to BH3 Domains

**DOI:** 10.1371/journal.pone.0003038

**Published:** 2008-08-22

**Authors:** Claire Grills, Nyree Crawford, Alex Chacko, Patrick G. Johnston, Francesca O'Rourke, Dean A. Fennell

**Affiliations:** 1 School of Applied Mathematics and Theoretical Physics, Queens University Belfast, Belfast, Northern Ireland; 2 Centre for Cancer Research and Cell Biology, Queens University Belfast, Belfast, Northern Ireland; 3 Northern Ireland Cancer Centre, Belfast, Northern Ireland; Tel Aviv University, Israel

## Abstract

**Introduction:**

The molecular mechanism underlying mitochondrial BAK activation during apoptosis remains highly controversial. Two seemingly conflicting models have been proposed. In one, BAK requires so-called activating BH3 only proteins (aBH3) to initiate its conformation change. In the other, displacement from inhibitory pro-survival BCL-2 proteins (PBPs) and monomerization of BAK by PBP selective dissociator BH3-only proteins (dBH3) is sufficient.

**Methodology/Principal Findings:**

To better understand the kinetic implications of these conflicting but highly evidence-based models, we have conducted a deterministic, dynamical systems analysis to explore the kinetics underlying the first step of BAK activation, as a non-linear reaction system. We show that dBH3 induced BAK activation is efficient, even in the absence of aBH3s, provided constitutive interaction of PBPs with open conformation BAK occurs in an adenoviral E1B 19K-like manner. The pattern of PBP expression robustly predicts the efficacy of dBH3s.

**Conclusion:**

Our findings accommodate the prevailing BAK activation models as potentially coexisting mechanisms capable of initiating BAK activation, and supports a model based approach for predicting resistance to therapeutically relevant small molecule BH3 mimetics.

## Introduction

Resistance to apoptosis is a hallmark of cancer and a pivotal factor underlying resistance to systemic anti-cancer therapy. Multidomain proapoptotic BCL-2 family proteins BAX and BAK are genetically redundant tumour suppressors and central regulators of apoptosis [1], [2]. BAK is a zinc regulated protein, and is constitutively localized to the outer mitochondrial membrane [3]–[5]. At least three steps are involved in BAK activation. The first step, involves a conformation change associated with exposure of the N-terminus. The second involves deep insertion into the outer mitochondrial membrane at the C terminus [6], and the third, oligomerization into a complex of as yet unknown stoichiometry leading to outer membrane permeabilization [7]. BAK auto-activation may drive this reaction forwards once initiated [8]. BAK oligomers cause mitochondrial outer membrane permeabilization (MOMP) by an unknown mechanism, leading to release of apoptogenic factors and activation of caspase dependent and independent events that in parallel, promote cell death. Once initiated, BAK mediates loss of the mitochondrial membrane potential that is required for oxidative phosphorylation, a reduction in cellular ATP level, and caspase independent cell death. Feedback mechanisms driven by caspases following MOMP also inhibit electron transport, ensuring cessation of respiration. Consequently, BAK activation when initiated causes a series of irreversible events that commit the cell to death.

BAK is activated by a subclass of proapoptotic BCL-2 proteins which share an amphipathic alpha helical BH3 domain (BH3-only proteins) [2], [5]. However, there currently exists considerable controversy as to how this activation occurs. Two seemingly irreconcilable models have been described. In the agonism model, a subclass of activator BH3-only proteins (aBH3s) comprising BID, BIM and arguably PUMA, interact with a putative activation binding site analogous to BAX [9], [10], leading to a conformation change and oligomerization [11]–[13]. Such activators may be constitutively bound to mitochondrial pro-survival BCL-2 family proteins such as BCL-2, or MCL-1. Under such conditions, described as “priming for death”, a second class of dissociator BH3-only proteins such as BAD or NOXA (dBH3s) can release activators to engage BAK [2], [14], [15]. This hierarchical BH3 regulation may underlie the activity of such small molecule dissociator BH3 mimetics such as ABT737 [15] or obatoclax [16]. It is the selectivity of dBH3s for their recognized pro-survival BCL-2s that determines BAK activating efficacy [17]. For example, coordinate restraint of BAK by BCL-XL and MCL-1 can be de-repressed by BAD and NOXA together, but not individually [18].

BAK is neutralized by BCL-2, BCL-XL, MCL-1 or VDAC2 [19], [20] and can be activated by the small molecule BAD BH3 mimetic ABT737, in the absence of aBH3s [21], [22]. This has led to the hypothesis that direct aBH3 dependent agonism is not essential for BAK activation, but that antagonism of pro-survival BCL-2 family proteins alone is sufficient [21]. This is the second conflicting model of BAK activation.

Pure agonism versus de-repressor models reflect contrasting thermodynamic representations of BAK regulation. In the agonism model, BAK's requirement for ligand driven conformation change suggests an intrinsic energy barrier or activation energy that prevents spontaneous activation, and must be surmounted. This is facilitated by the agonist in a catalytic-like manner. A corollary of this model is that BAK should be capable of residing in a stable inactive monomeric conformation, until bound by its agonist ligand. In direct contrast, the de-repressor model suggests that BAK will spontaneously unfold its N-terminus unless a constitutive repressor is bound. Release of BAK by dBH3s will then cause its activation.

Because these scenarios are in conflict, we have employed a deterministic mathematical modelling strategy to explore the concentration-dependent effects of aBH3 and dBH3s alone or in combination, on the maximum rate of BAK activation. Our findings suggest that both the agonism and dissociation models reflect valid and potentially coexisting mechanisms for BAK activation, provided that strict constraints are applied.

## Results

### Mitochondrial BAK activation by an aBH3 domain

**Figure 1:**
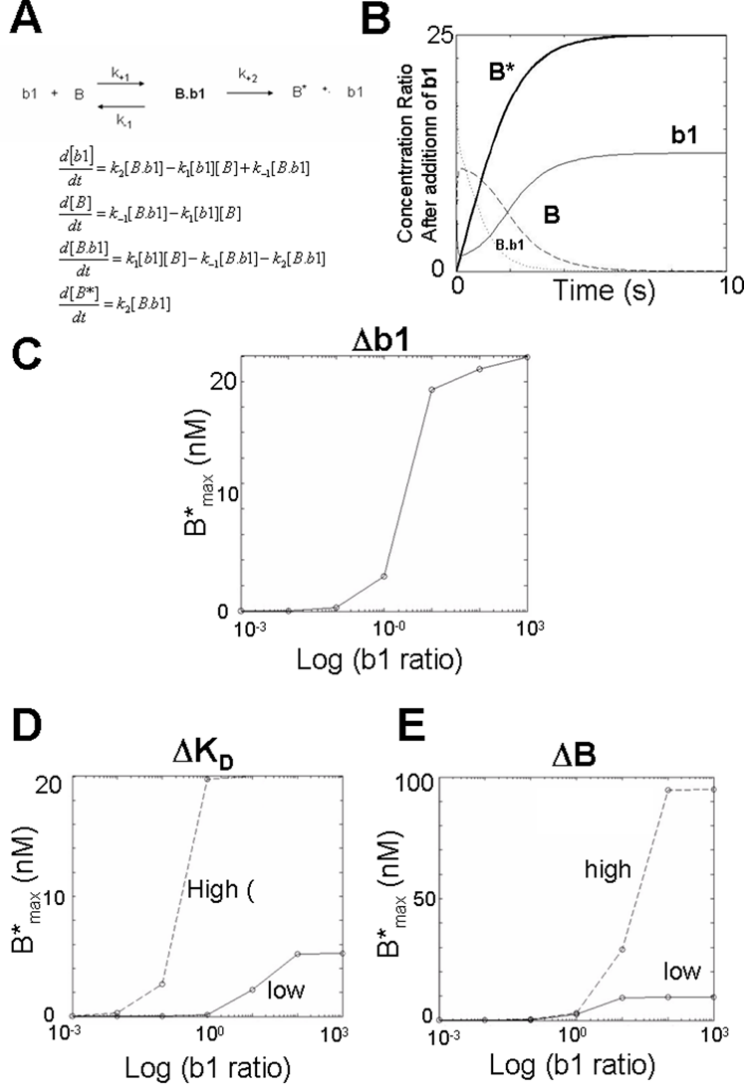
Modelling the outer mitochondrial membrane bimolecular collision between and activating BH3 peptide and BAK. A) Reaction scheme showing the interaction between b1 and B leading to formation of a transition complex, and its dissociation to free open BAK (B*) and recycled b1. Below is the corresponding system of differential equations corresponding to this system. B) Graph showing the timecourse of changing concentration of species following an instantaneous concentration jump in b1. Note, B* tends to a plateau, B*. C) Concentration response relationship for log b1 ratio vs B* as an iterative solution to the corresponding system of equations. D) Graphs showing the dependency of b1 potency on the affinity of b1 for B E) Graph of log b1 ratio against B*; the concentration of B has a significant influence on the maximum activity of b1.

The solutions for the simplest BAK activation model involving a bimolecular reaction between b1 (aBH3 domain) and B (BAK) to yield B* (open conformation BAK), is defined by four linear first order differential equations (figure 1A), and yields graphs of the change B, b1, b1.B, and B* with time (figure 1B). B* exhibits a rapid initial rise and tends to a plateau corresponding to the maximum output, B*_max_. Accordingly, B is consumed by the reaction as B* is formed. The transition complex B.b1 exhibits a transient increase in level, which reduces to zero as it converts to B*.b1 and dissociates to B* and b1. Free b1 initially declines in level as it is consumed into B*.b1, but increases upon dissociation from its complex with B*, becoming free again to participate in further reactions until all available B is been converted to B* (figure 1B).

Solving the dynamical system with seven iterations over a six log fold range of b1 ratio, yields different values of B_max_ and a sigmoidal concentration response (figure 1C), such that B*_max_ increases to a limit. The ratio of b1 is given by the ratio of 0.001, 0.01, 0.1, 1,10,100 or 1000 to the initial value 1×10^9^ M and is therefore dimensionless. The concentration-response curve is estimated by fitting the logistic function, and yields parameter estimates of M = 19.48 (95%CI = 18.62 to 20.34) , λ = 2.95 (95%CI = 2.22 to 3.92), and p = 1.71 (95%CI = 1.30 to 2.11). The logistic function provides a good fit to the log b1 concentration-B*_max_ curve, with R^2^ = 0.997

A decrease in the affinity of b1 for B, ΔK_D_ (reducing from a high value of 100 to 10) produces a corresponding decrease in the potency of b1, such that both the maximum achievable output of the system, and ratio producing half maximal output, are significantly reduced. The plateau parameter estimated by regression reduces from 20 (95%CI = 19.82 to 20.18 for K_D_ = 10×10^−9^ M) to 5.63 (95%CI = 5.12 to 5.42 for K_D_ = 0.1×10^−9^ M). The estimated λ reduced from 12 (95%CI = 10.71 to 13.47) to 0.2 (95%CI = 0.17 to 0.24) with increasing affinity. This is consistent with the dramatic increase in BH3^BID^ efficacy observed experimentally, when the alpha helix is stabilized by olefin metathesis and all-hydrocarbon stapling [13], [23]. An increase in the amount of available B in the reaction (ΔB), from 10 to 100 also results in a corresponding change in B*_max_ (figure 1E). The M parameter estimated by regression increases from 9.50 (95%CI = 9.28 to 9.71 for B = 10) to 95 (95%CI = 91.05–99.70 for B = 100). Consequently, the rate of B* generation by b1 is critically dependent on the amount of B in the system consistent with the observed loss of efficacy of BH3 domains in the absence of BAK (and BAX) [2].

### Regulation of B* kinetics by Pro-survival BCL-2 proteins and reversal by dBH3s

**Figure 2 pone-0003038-g002:**
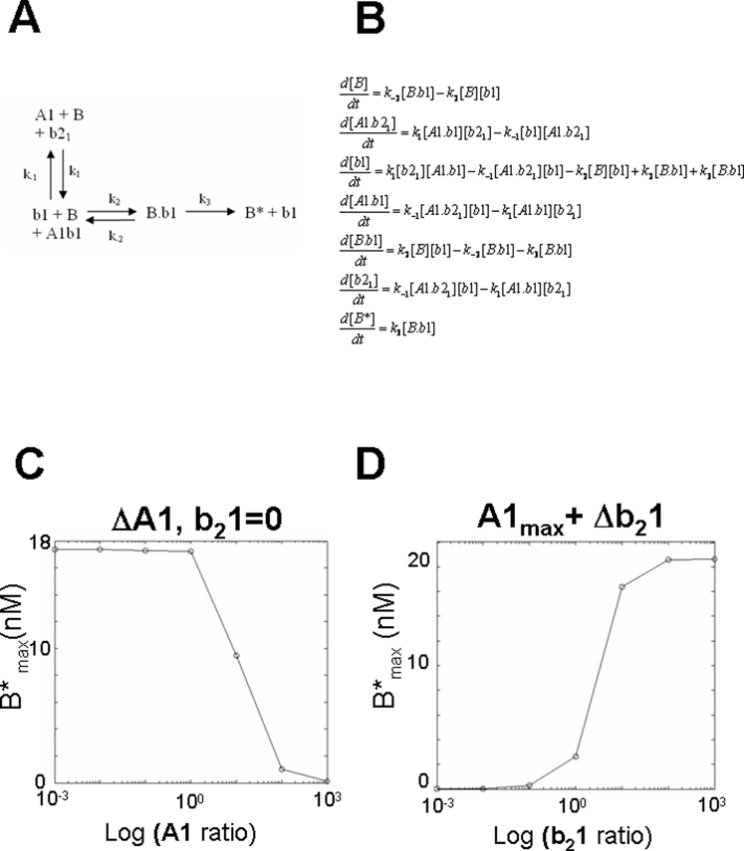
Modelling the antagonism of an activating BH3 domain by a single prosurvival BCL-2 protein, and its de-repression by addition of a dissociating BH3 domain. A) Reaction scheme showing the interaction between A1, b_2_1, b1 and B. In this reaction, A1 binds to b1. B) The corresponding system of differential equations corresponding to this reaction scheme. C) Graph showing the reduction in B* with increasing A1 ratio. D) Concentration response relationship for log b_2_1 ratio vs B* as an Iterative solution to the corresponding system of equations.

Introducing a single prosurvival BCL-2 protein (A1) results in the reaction scheme (where the A1 targeting dBH3, b_2_1 = 0) and system of differential equations shown in figures 2A and 2B respectively. As A1 ratio increases over a 6 logfold range, B*_max_ reduces, exhibiting bistability with a steep fall in B*_max_ production towards zero at higher A1 concentrations (figure 2C). Therefore, the presence of A1 per se does not necessarily suppress b1 driven B* formation, however, above a certain threshold level, the production of B* will rapidly switch off, causing complete resistance to b1. This property is consistent with the rheostat model originally described in relation to BAX/BAK by Korsmeyer and colleagues [24].

At maximum A1 ratio (A1_max_ = 10), this is associated with complete suppression of B* production. However when the dBH3 b_2_1 is introduced, and increases, B* production is efficiently de-repressed, achieving B*_max_ close to that in the absence of A1 at b21_max_ = 10 (plateau parameter estimate, M = 19.48 (95% CI = 18.62 to 20.34 for A1 = 0, b_2_1 = 0) compared with M = 18.61 (95%CI = 18.41 to 18.82 for A1 = 10, b_2_1 = 10, figure 2D). Accordingly, where a dominant prosurvival protein is expressed above critical threshold concentrations required to efficiently suppress B*_max_, a dBH3 domain targeting A1, can de-repress the system allowing near full activation of B when the stoichiometric ratio of b_2_1:A1 approaches reaches 1.

### BAK antagonism by two prosurvival BCL-2 family proteins requires a second type of dBH3 to enable activation

**Figure 3:**
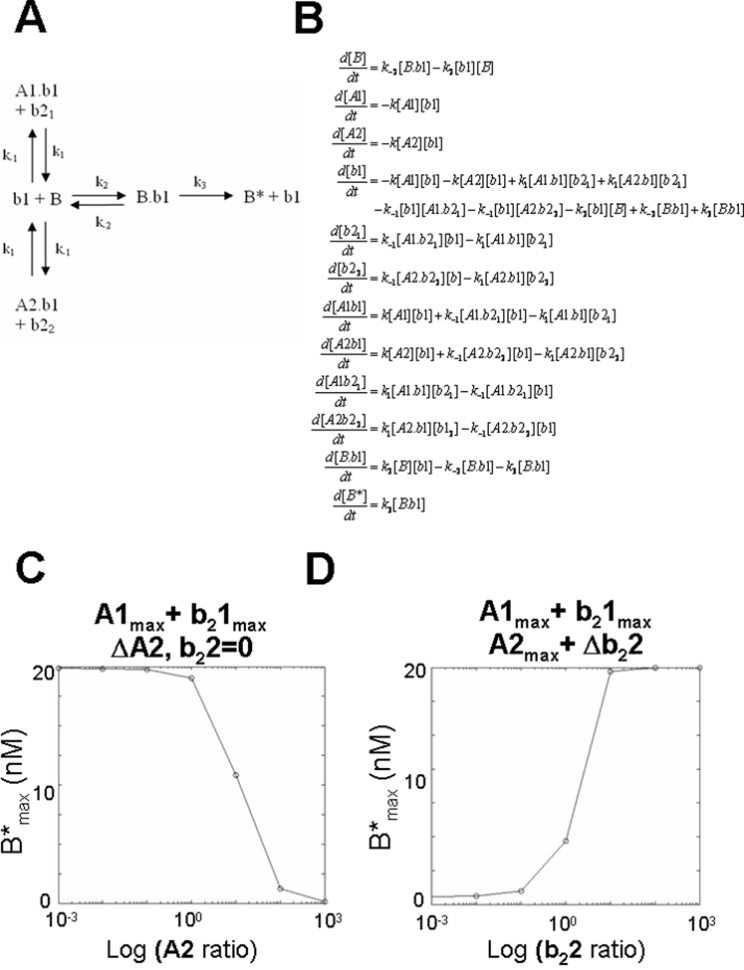
Modelling the antagonism of an activating BH3 domain by a second prosurvival BCL-2 protein, and its de-repression by addition of a second dissociating BH3 domain. A) Reaction scheme showing the interaction between A1, A2, b_2_1, b_2_2, b1 and B. In this reaction, A1 and A2 bind to b1. B) The corresponding system of differential equations corresponding to this reaction scheme. C) Graph summarizing the iterative numerical solutions of the corresponding system of differential equations, showing the reduction in B* with increasing A2 ratio at maximum A1 and b_2_1. D) Concentration response relationship for log b_2_2 ratio vs B* as an Iterative solution to the corresponding system of equations in the presence of maximum concentration of A1, A2 and b_2_1.

Although the suppressing effect of A1 on B*_max_ is efficiently reversed by b_2_1, introducing a second prosurvival BCL-2 family protein, A2 which binds b1 but does not interact with b_2_1 results in the reaction scheme and system of differential equations shown in figures 3A and 3B. With increasing A2 ratio in the presence of A1_max_ and b_2_1_max_, B*_max_ reduces, albeit with a less steep decline compared to that of A1 alone (slope parameter, p = −1.23 (95%CI = −1.31 to −1.24, compared with p = −1.53 (95%CI = −2.10 to −0.96 for A1_max_,A2_max_,b21_max_. However, complete suppression is still achieved as A2 tends towards a maximum effective ratio (A2_max_ = 10), as shown in figure 3C. Introduction of a second BH3 domain (b_2_2) which interacts selectively with A2, can efficiently de-repress B* formation in the presence of A1_max_/b_2_1_max_ and A2_max_. The maximum output achievable by b_2_1 (at b_2_1_max_ = 10) is the same as that even in the absence of either A1,A2,b_2_1 or b_2_2 (M = 20.1 95%CI = 19.84 to 20.17)

### BAK activation kinetics in the absence of aBH3

**Figure 4:**
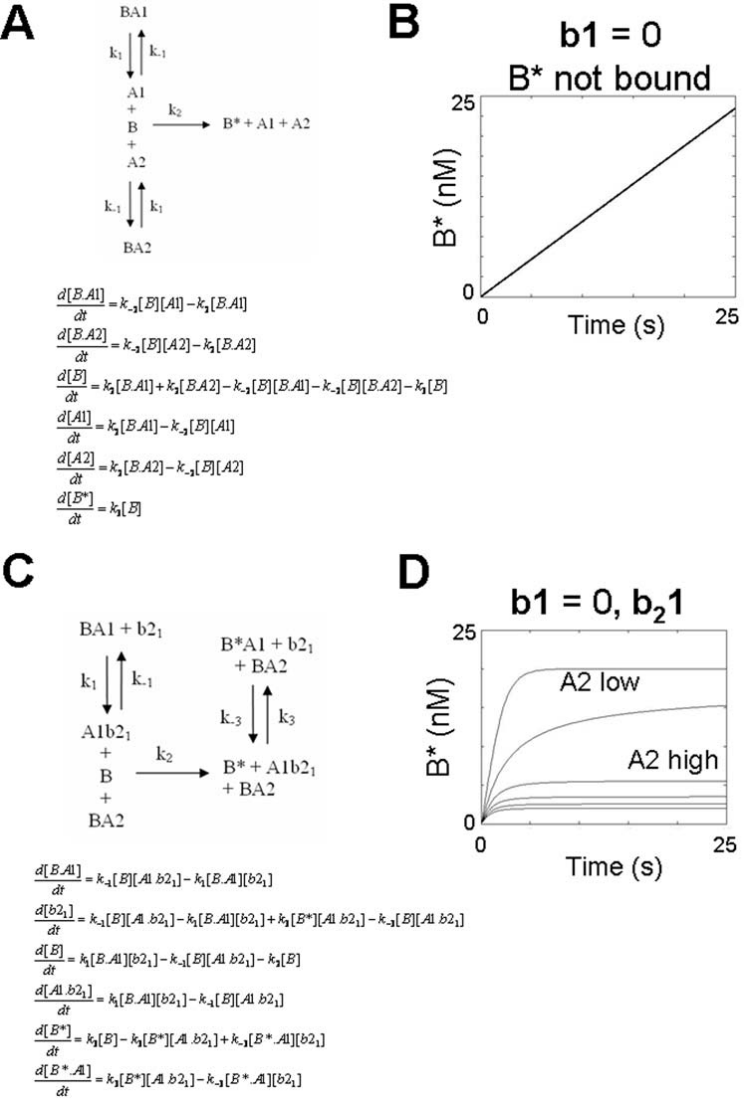
Activation of BAK in the absence of an activating BH3 domain. A) Reaction scheme showing the coordinate antagonism of B* by A1 and A2 in the absence of b1. Binding to B only is shown. The corresponding system of differential equations are shown below. B) Plot of B* showing the inherent instability of this system over time. C) Modified reaction scheme with b1 = 0 in which B* is now bound by A1. The system includes b_2_1 and A2. D) As A2 increases, B* formation is increased in the absence of b1.

Recent genetic evidence supports an activation model for BAK which does not require the presence of aBH3s (BID, BIM or PUMA) [18], [21]. This suggests that overcoming an activation energy for conformation change of BAK is not required, rather, that activation involves de-repression and spontaneous transit to a lower energy state. In the dynamical systems model, B therefore requires restraint by PBPs A1 and A2 , which can co-ordinately bind B into two kinetic compartments, namely B.A1 and B.A2 (figure 4A). However, since the transition B→B* is assumed to be essentially irreversible, and the interactions of B with A1/A2 are assumed to be reversible, this model is highly unstable with B* forming spontaneously, relatively slowly over time (figure 4B).

**Figure 5:**
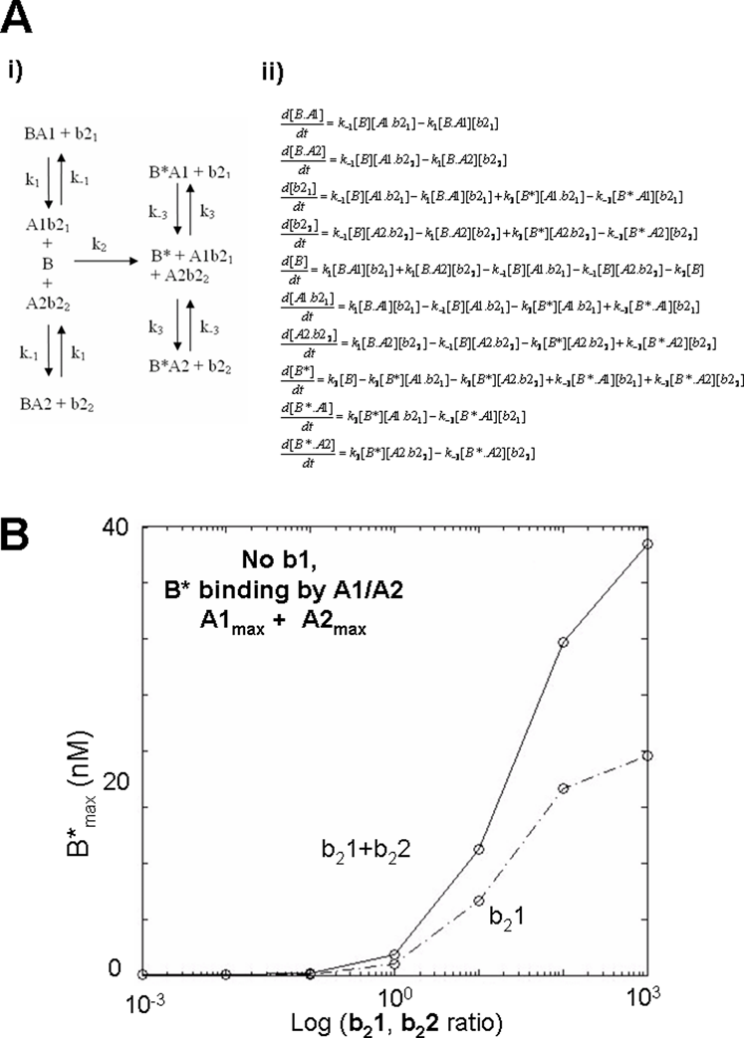
Effect of two dBH3 domains: Open BAK restrained by two-prosurvival BCL-2 proteins in the absence of a aBH3 peptide. A) Reaction scheme showing the interaction between A1, A2, b_2_1, b_2_2, and B in the absence of b1. In this reaction, A1 and A2 bind to b1, B and B*. B) The corresponding system of differential equations associated with this reaction scheme. C) Graph of the iterated solutions of the corresponding system of equations at varying b1 showing the relative effects of b_2_1 with or without simulanteous variation in b_2_2 across a three log-fold range, on B*.

Adenoviral E1B 19K and BCL-2 have been reported to interact with BAK in its open conformation [8]. In the dynamical system where B and B* interact with A1/A2 (figure 4C), the system is stable, and A1/A2 can suppress free B* formation (figure 4D). Under these conditions, de-repression of bound B and B* can be achieved by addition of b_2_1 with or without b_2_2 (figure 5A). Together b_2_1 and b_2_2, when varied with the same concentration ratio, are more effective in freeing B* than b_2_1 alone leading to a higher B*_max_ at increasing concentration ratio (M = 20 95%CI = 19.98 to 20.02 for b_2_1 alone), compared with M = 40 (95%CI = 39.80 to 40.92 for b_2_1 and b_2_2, figure 5B).

### BAK activation by an aBH3 is enhanced by dBH3s when PBPs inhibit open BAK

**Figure 6:**
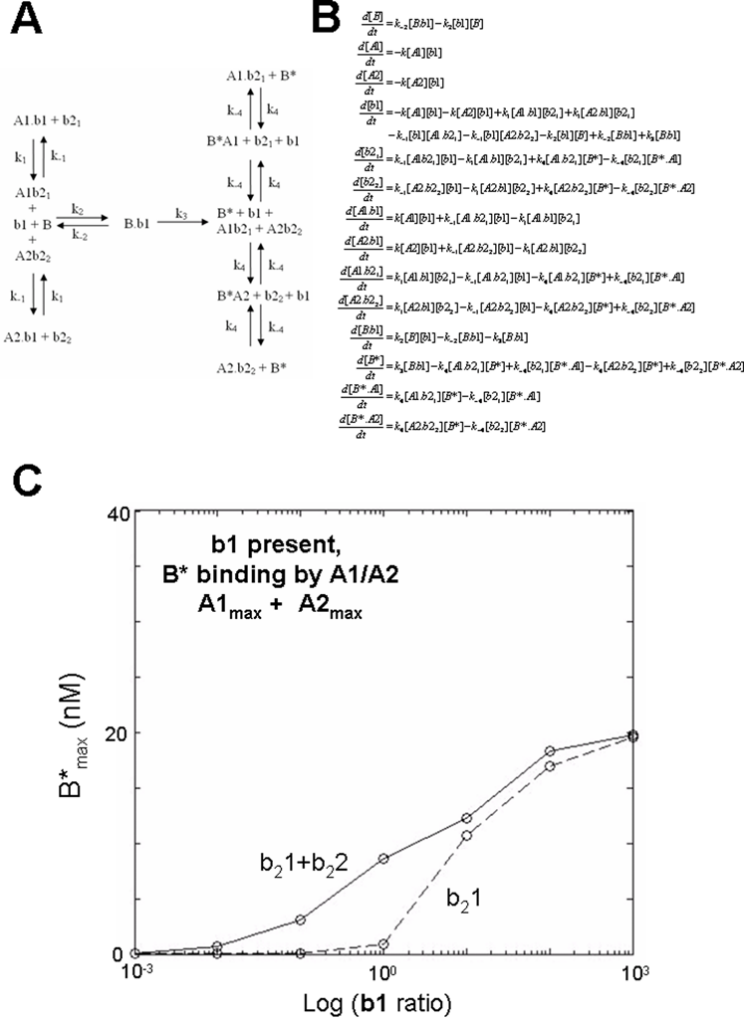
Effect of two dissociating BH3 domains: Open BAK restrained by two-prosurvival BCL-2 proteins in the presence of an activating BH3 peptide. A) Reaction scheme showing the interaction between A1, A2, b_2_1, b_2_2, B in the presence of b1. In this reaction, A1 and A2 bind to b1, B and B* and are at maximum concentration of 10 nM. B) The corresponding system of differential equations associated with this reaction scheme. C) Graph of the iterated solutions of the corresponding system of equations, showing log b1 ratio versus B*; b_2_1 effectively derepresses the system enabling B* output, and addition of b_2_2 further enhances b1 potency, causing a shift of the concentration-response curve to the left.

Since binding of B* to A1 with or without A2, is essential for stable suppression of B* in the absence of b1, this mechanism can be incorporated into a dynamical system that includes b1 driven opening of B, with B* binding by A1/A2 (figures 6A and 6B). Under these conditions, b1 in the presence of b_2_1 results in activation of B* even though A2 is present, however the maximum output of the system is the same as for b_2_1 activity in the absence of b1 shown in figure 5C (ie. M = 19.02 95%CI = 16.66 to 21.37 in the presence of b1). Note, B* production is zero when b_2_1 = 0 and b_2_2 = 0 in this system since spontaneous B* is not assumed to occur.

When b_2_2 is added to the system, b1 efficacy is enhanced with a decrease in the EC_50_ with λ reducing from 8.4 (95%CI = 4.86 to 14.63 for b_2_1 only), to 3.12 (95%CI = 0.62 to 15.80 for b_2_1 plus b_2_2). In contrast, the maximum output of the system remains approximately constant with M = 21.9 (95%CI = 15.65 to 28.04 for b_2_1 plus b_2_2) compared with M = 19.02 (95%CI = 16.66 to 21.37 for b_2_1 only). Accordingly, b_2_1 and b_2_2 enhance b1 efficacy, resulting in left shift of the concentration response relationship, but with reduced maximum B* production, compared with the system in which b1 is absent.

### Maximum BAK opening by dBH3s is limited by the presence of the aBH3 in a unified model

**Figure 7:**
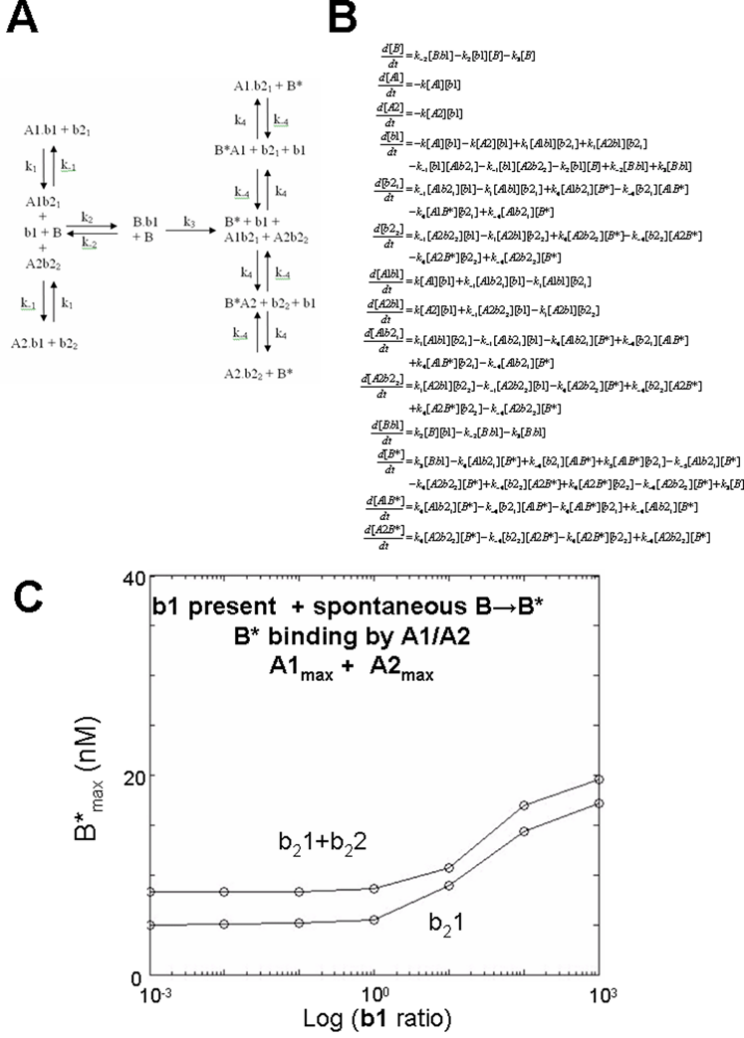
Unified model incorporating both b1 driven and spontaneous activation of B. A) Reaction scheme showing the interaction between A1_max_, A2_max_, b_2_1, b_2_2, B in the presence of b1. In this reaction, A1 and A2 bind to b1, B and B* and are at maximum concentration of 10 nM. B bifurcates in its activation with equal probability leading to either spontaneous activation or b1 driven activation. B) The corresponding system of differential equations associated with this unified reaction scheme. C) Graph of the iterated solutions of the corresponding system of equations, showing log b1 ratio versus B* for b_2_1 alone or b_2_1 plus b_2_2.

As previously shown, in order for dBH3s to be able to generate B* in the absence of b1, spontaneous activation of B* must be assumed, with constitutive binding of A1/A2 to B*. Accordingly formation of B* would occur via two simultaneous pathways, one driven by b1, the other by derepression of spontaneously formed B*. Since the prevailing models of BAK activation suggest that each are equally possible, both activation pathways can be combined in a unified model (figure 7A and 7B).

The plot of log b1 ratio against B*_max_ in the presence of b21 (figure 7C) shows that b_2_1_max_ alone in the presence of A1_max_ and A2_max_ can induce the low level production of B* when b1 = 0.001 (the level of B*_max_ is 5.0, 95%CI = 4.63 to 5.30), however addition of b1 across a 6 logfold range, increases B* production in a concentration dependent manner tending to a maximum (M = 17.84, 95%CI = 16.88 to 18.80). When b_2_2 is added to b_2_1, B* production by b1 is increased slightly , leading to a higher maximum output (M = 19.9, 95%CI = 19.94 to 20.01). The initial level of B* production (at b1 = 0.001) is also higher than for b_2_1 alone (8.9, 95%CI = 8.28 to 8.30). The output of this unified system is similar to that of b_2_1/b_2_2 in the *absent b1* model shown in figures 4–5, but greater than the agonistic models described in figures 1–3. From the unified model, it is clear that b_2_2 and b_2_1 are required to enable maximum potency of b1 when both A1_max_ and A2_max_ are present. Somewhat paradoxically however, the presence of b1 limits the maximum output achievable by the dynamical system by around 50% compared with the *absent b1* model (figures 4 and 5).

## Discussion

We have used dynamical systems analysis to explore the kinetics of mitochondrial BAK activation in the presence or absence of aBH3s, in order to address the question of how efficiently dBH3s induce apoptosis at the level of the outer mitochondrial membrane. Mathematical modelling enables deeper insight and understanding of complex dynamic systems [1], [2]. However, there have been no attempts to date to model mitochondrial membrane resident BAK in the context of multiple PBPs nor multiple dBH3s, which are both biologically relevant. *In silico* modelling has been reported in relation to BAX , however these studies were limited to considerable simplification of the interactions between one PBP, aBH3 and BAX, and have attempted to incorporate both mitochondrial translocation of BAX/aBH3 and outer mitochondrial permeabilization events, both of which are poorly defined at the molecular level [25], [26]. Furthermore, BAX/BAK are resident in the endoplasmic reticulum as well as the mitochondrial compartment, and probably behave as interacting dynamical systems. For example, the BH3 only proteins BIK triggers ER BAK leading to ER-mitochondrial interactions and apoptosis [27], [28].

In reality, the interplay between BCL-2 family proteins is more complex than that which has been previously mathematically modelled, even at the level of the mitochondrial membrane alone, by virtue of their heterogeneity. Cancer cell models commonly harbour constitutively expressed BAK and multiple PBPs (these may include BCL-2, BCL-XL, MCL-1, A1 and BCL-W coexpression in the case of non-small cell lung cancer – data unshown). Furthermore, so-called priming for death involves mitochondrial co-expression of BH3s (aBH3s and dBH3s) which have translocated constitutively and are neutralized by PBPs [29], [30]. Membrane bound BAK activation and engagement with PBPs and BH3s takes place in a single spatial compartment, potentially enabling more robust and accurate models of BAK activation by a BH3 domain peptide to be defined. We have therefore focused on a reduced but experimentally testable system that explores the interaction of BH3 domains at the isolated mitochondrial surface, which has been shown to conserve functional BAK.

We demonstrate that the *within-membrane* interactions of mitochondrial BCL-2 proteins and BH3 domains, although complex, are amenable to this mathematical modelling approach. A considerable body of evidence has amassed implicating aBH3 domains as being capable of triggering multidomain BCL-2 family protein activation [11], [12], [17]. Putative activating residues in BAX have been proposed through mutational analysis [9] and stabilization of BH3 domains using all hydrocarbon stapling has demonstrated the activating ability of BID BH3 domain [13]. The ability of aBH3s to drive BAK conformation change and oligomerization strongly suggests that as yet unidentified trigger residues also resides in BAK. Because mitochondria can be isolated, and respond to aBH3 peptide domain in a manner observed *in vivo* [11], [12], our model incorporates a rapid (near instantaneous) concentration jump of aBH3, leading to initiation of the reaction leading to B*. This is consistent with pharmacological exposure in a cell free system, and is relevant to modelling the action of small molecule BH3 peptidomimetics [15].

Protein-protein crosslinking studies in isolated mitochondria clearly identify constitutively monomeric BAK in the outer mitochondrial membrane [8]. BMH internally crosslinks BAK cysteine residues 14 and 166, leading to a fast mobilizing band on SDS PAGE. This fast band reflects within-membrane, closed conformation BAK, and is lost in the presence of BID BH3 peptide, consistent with unfolding and activation at the level of a monomeric species. The existence of closed conformer BAK in the outer mitochondrial membrane of healthy isolated mitochondria also implicates a constitutively left-shifted equilibrium strongly towards a closed/inactive conformation. Therefore, a requirement for constitutive PBP repression alone in this compartment is unlikely. If BAK can reside in the outer mitochondrial membrane as an inactive monomer, collision with an aBH3 would be required for activation. This model is entirely inconsistent with BAK requiring constitutive repression only to prevent its activation as suggested from genetic studies [18], [21]. Conversely, robust genetic evidence has confirmed that BAK activation can proceed in the absence of known aBH3s, suggesting that in some systems, constitutive BAK-PBP interaction is necessary to prevent its activation.

This raises the important question of how two seemingly contradictory models can be reconciled. Dynamical systems analysis provides a powerful tool to capable of providing important insights to explain these experimentally observed phenomena. Our results strongly suggest that stable complexes of BAK can only occur in the open conformation (B* as opposed to B), and that disruption of complexes with PBPs will yield free B*, which is assumed to be capable of further activation (deep membrane insertion, oligomerization, and autoactivation). B* binding is observed for adenoviral E1B19K and BCL-2 . A central paradox in this model, is how B* forms in the first place, when no aBH3s are present (ie. in genetic knockouts). This may be explained in at least two ways. Firstly, unknown death signals may be capable of driving BAK conformation change in the absence of the known aBH3s BID, BIM and possibly PUMA. Secondly, conversion of B to B* may happen spontaneously, albeit with much lower probability compared with aBH3 driven conversion. In the absence of aBH3s therefore, pro-survival BCL-2 proteins would therefore provide an efficient safety mechanism for blocking BAK activation after it has undergone its conformation change.

An important question relates to the fate of B* upon release from its complex with either A1 and/or A2 by b21 and/or b22. B* can autoactivate other B molecules to form oligomers. Furthermore, b1 which is also freed from A1 and/or A2 is then capable of causing ballistic conversion of B to B* in the outer mitochondrial membrane. What is clear from experimental studies, is that the pharmacological characteristics of BH3 domains differ significantly. Therefore, BH3^BAD^ and BH3^NOXA^ although able to sensitize BH3^BID^, does not mimic this aBH3 [11], [12].

The systems of equations solved for the reaction schemes described here, consider only the first step in the activation of BAK. Free open conformation BAK may nucleate other closed B molecules, through an autoactivation [8]. Auto-activation would therefore be expected to dramatically increase the kinetics of free B* formation. Furthermore, auto-activation may result in nucleation of newly activated Bs into a multimeric B* complex following homo-oligomerization [7]. The mechanisms and kinetics of BAK oligomerization are poorly understood. MOMP can occur within minutes of exposure to BH3 peptide or full length protein, however the minimal stochiometry of the molecular apparatus responsible for MOMP is as yet undetermined. The electrophysiologically detectable mitochondrial apoptosis channel comprises BAX and BAK, and may require other proteins for assembly [31], [32]. For example, a recent report implicates the TOM complex in the process of MOMP. Assembly of the outer membrane pore may proceed ballistically and without limit, leading to the observale BAX/BAK clusters previously reported [7].

The modelling described, assumes stable levels of the interacting molecules over time. It is known that antiapoptotic BCL-2s such as MCL-1 can undergo marked changes due to proteosomal degradation [21] following its dissociation. As such, the influence on b_2_2 on A2 could in some systems increase the non-linearity of the system due to alterations in A2 level with time as well as dissociation from B*, B and b1.

Our results demonstrate that the selectivity of binding of BH3 only proteins to their PBP counterparts in the outer mitochondrial membrane, clearly has a major impact on B*. The ability of dBH3s and small molecule BH3 peptidomimetics to free B* should therefore be associated with significant proapoptotic pharmacological activity, in a manner that is not significantly influenced by the expression of aBH3s in cancers. Resistance to dBH3 domains or peptidometics will therefore depend on the expression pattern of PBPs. The shape of the b21/b22 concentration response curves suggests that A1 and or A2 expression above a critical level is essential to efficiently suppress B*. This has implications for resistance biomarker screening. For example, the BH3 peptidomimetic ABT737 is inhibited by overexpression of MCL-1, consistent with its BAD like binding selectivity to BCL-2/BCL-XL/BCL-W. However, our modelling predicts that MCL-1 levels below a threshold of expression and not MCL-1 expression *per se*, should dictate resistance to BAD-like peptidomimetic induced apoptosis.

We have examined the predicted behaviour of unified system in which both spontaneous B→B* generation occurs resulting in a pool of A1/A2 inhibited B*, and also b1 driven B→B*. In this model, as expected b1 achieves a concentration dependent increase in B*_max_, however, somewhat paradoxically, the presence of b1 limits the magnitude of B_max_ that is achievable in the unified model. Cancer cells have been shown to spontaneously process aBH3s, which are neutralized at the mitochondrial surface accounting for the so-called priming for death phenomenon. With respect to opening of BAK, which alone is not sufficient to induce MOMP, b1 is predicted from the unified model to reduce the amount of B*. One might anticipate that the selection pressure that leads to the antiapoptotic phenotype in cancer might reduce, not increase the mitochondrial priming for death with aBH3 tumour suppressors. The unified model, provides a potential explanation for this experimental observation and suggests that this phenomenon effectively limits the maximum BAK activation achievable by dBH3 induced B*_max_. Conversely, elimination of primed aBH3s, might be expected to enable higher B*_max_ in response to dBH3s. Nevertheless, where b1 is present in a system, addition of dBH3s will potentiate b1 mediated B* activation, consistent with experimental observation and therefore strongly supports the use of BH3 peptidomimetics as potential as anticancer agents.

In summary, dynamical systems analysis of BAK activation reconciles and supports a general model in which the interplay between BAK, and multiple PBPs determine susceptibility to dBH3 domains (and therefore mimetics such as ABT-737 and GX15-070), in both the presence or absence of an aBH3. As such, this approach has implications for better understanding of the complex molecular mechanisms that underlies BCL-2 family addiction, the implications of mitochondrial priming for death, as well as critical factors governing sensitivity or resistance to BH3 peptidomimetics now entering the clinic.

## Materials and Methods

### BAK activation by a BH3 agonist

A one dimensional deterministic system was established to model the rate of BAK activation in the mitochondrial outer membrane according to the agonism model. The diffusion path of BAK is constrained to the plane of the outer mitochondrial membrane where it has been proposed to directly interact with aBH3 domains. When an aBH3^BID^ is applied to isolated state IV mitochondria, BAK is rapidly converted from a closed monomeric form to an open form, as evidenced by loss of an internally crosslinked species due to lack of bismaleimeidohexane crosslinking between cysteines 14 and 166 [8]. Applying the the law of mass action to the bimolecular collision of an aBH3 domain (b1) with BAK, hereafter referred to as B, enables the forward reaction rate associated with the first step of BAK activation (B*) to be determined. It is assumed that an instantaneous b1 concentration jump initiates formation of B*, consistent with addition of an exogenous aBH3 or dBH3 domain peptide to isolated mitochondria. B is assumed to be constitutively monomeric, immediately prior to collision with b1.

B and b1 are assumed to form a transition complex (B.b1). B then undergoes transition to its open conformation, B*. The complex B*.b1 is assumed to have a very short lifetime, reflected in observed “kiss-and-run” BID and BAX [13]. It is therefore assumed that the transition B.b1→B*.b1 is sufficiently rapid to justify simplification by encompassing B.b1→B*.b1 into the single reaction B.b1→B* + b1, with the associated *conformation change* rate coefficient k_+2_. It is assumed that recycling of free b1 follows dissociation of B.b1, enabling its re-use in subsequent interactions and ballistic B* generation.

### Modelling multiple Pro-survival BCL-2 protein interactions and their antagonism

Prosurvival BCL-2 family proteins (PBPs) A1 or A2 interact with b1 preventing its binding to B to yield B*[30]. PBPs can also interact with B and B* [8], [30]. In turn, A1 and A2 exhibit selective interactions with the dBH3s b_2_1 and b_2_2, which displace b1 from A1/A2 to form the stable complexes b_2_1.A1, and b_2_2.A2 respectively. The interaction of A1 with b_2_1 is analogous to to the selective binding of BH3^BAD^ to mitochondrial BCL-2, BCL-XL or BCL-W, whereas the A2 interaction with b_2_2 corresponds to the interactions of MCL-1 or A1 with with BH3^NOXA^. For the purposes of simplification, the variables A1 and A2 can be considered to represent functionally redundant pools of mitochondrial PBPs with identical stoichiometry, affinities and effects on B* kinetics, whereby, A1 corresponds to BCL-2, BCL-XL, BCL-W and A2 corresponds to MCL-1/A1.

### Modelling BAK activation in the absence of an agonistic BH3

The rate of B* generation under conditions where b1 = 0, corresponds to the recently reported double BID/BIM knockout plus PUMA knockdown model system [18], [21]. By removing b1 from the system, the effect of b_2_1 with or without b_2_2 on expressed A1, with or without A2 can be examined, solving for B*.

### Computational Modelling

It is assumed that BAK activation kinetics can be analyzed using classical reaction kinetics and application of the law of mass action. The variables B, A1, A2, b_2_1, b_2_2 are initially constant until introduction of b1 as an instantaneous concentration jump. The reactants are assumed not to exhibit significant changes in concentration, which might be due to either synthesis or degradation over the timescales of interest. Systems of ordinary differential equations corresponding to different reaction schemes were solved numerically using MATLAB (Mathworks, Massachusetts) ODE solvers on personal desktop computer installed with the Linux operating system. For simplification, all equilibrium dissociation rate constants (ratio of association to dissociation rate constants) are set to 1×10^−9^ M. This reflects the order of magnitude of binding affinities for dBH3 domain-PBP interactions reported using surface plasmon resonance measured under *ex vivo* conditions [17] and novel BH3 peptidomimetics such as ABT-737 [15]. Initial concentrations of all reactants are assumed to be 1×10^−9^ M. The simplified conformation change rate constant k_2_ corresponding to combined B→B* transition and dissociation of B*.b1 is unknown but is assumed to be 1×10^−6^ S^−1^ corresponding to an efficient enzyme catalytic rate (http://doqcs.ncbs.res.in/).

In the dynamical systems described, the effect of varying the A1, A2, b_2_1 or b_2_2 ratio across a 6 logfold range [10^−3^, 10^3^] on the maximum output of the system (B_max_) are determined graphically, through iterative solving of the corresponding system at varying concentrations of b1 or other specified variable. The denominator of the concentration ratio is taken to be 1×10^9^ M for all reactants, the numerator being the logfold multiples of this value (10×, 100×, or 0.1×, 0.01× etc). As such, concentration-response relationships can be generated (over seven iterations), under conditions which correspond to the presence or absence of A1/A2 with or without b_2_1/b_2_2 in the presence or absence of b1. Where indicated, the effect of A1, A2 or b1 is estimated as the log concentration ratio producing half maximal reduction or increase in B* respectively (EC_50_) estimated by non-linear regression. The inverse logistic function is used to estimate EC_50_ for A1/A2 and corresponds to the equation:-
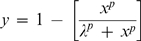
where λ estimates the EC_50_ and p is the Hill-slope of the curve; the logistic function for analysis of the b1 concentration response relationship is given by the equation:- 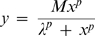
where M is the plateau estimator. Curve fitting employed the GraphPad Prism non-linear regression application (La Jolla). All parameter estimates from non-linear regression are shown with ± approximate 95% confidence limits.
